# Application of Mesenchymal Stem Cells During Machine Perfusion: An Emerging Novel Strategy for Organ Preservation

**DOI:** 10.3389/fimmu.2021.713920

**Published:** 2021-12-22

**Authors:** Jiale Li, Qinbao Peng, Ronghua Yang, Kunsheng Li, Peng Zhu, Yufeng Zhu, Pengyu Zhou, Gábor Szabó, Shaoyi Zheng

**Affiliations:** ^1^ Department of Cardiovascular Surgery, Nanfang Hospital, Southern Medical University, Guangzhou, China; ^2^ Department of Burn Surgery and Skin Regeneration, The First People’s Hospital of Foshan, Foshan, China; ^3^ Department of Cardiothoracic Surgery, Nanjing Drum Tower Hospital, Nanjing University Medical School, Nanjing, China; ^4^ Laboratory Animal Research Center, Nanfang Hospital, Southern Medical University, Guangzhou, China; ^5^ Department of Cardiac Surgery, Heidelberg University Hospital, Heidelberg, Germany; ^6^ Department of Cardiac Surgery, University Hospital Halle (Saale), Halle, Germany

**Keywords:** mesenchymal stem cells, machine perfusion, ischemia-reperfusion injury, organ preservation, transplantation

## Abstract

Although solid organ transplantation remains the definitive management for patients with end-stage organ failure, this ultimate treatment has been limited by the number of acceptable donor organs. Therefore, efforts have been made to expand the donor pool by utilizing marginal organs from donation after circulatory death or extended criteria donors. However, marginal organs are susceptible to ischemia-reperfusion injury (IRI) and entail higher requirements for organ preservation. Recently, machine perfusion has emerged as a novel preservation strategy for marginal grafts. This technique continually perfuses the organs to mimic the physiologic condition, allows the evaluation of pretransplant graft function, and more excitingly facilitates organ reconditioning during perfusion with pharmacological, gene, and stem cell therapy. As mesenchymal stem cells (MSCs) have anti-oxidative, immunomodulatory, and regenerative properties, mounting studies have demonstrated the therapeutic effects of MSCs on organ IRI and solid organ transplantation. Therefore, MSCs are promising candidates for organ reconditioning during machine perfusion. This review provides an overview of the application of MSCs combined with machine perfusion for lung, kidney, liver, and heart preservation and reconditioning. Promising preclinical results highlight the potential clinical translation of this innovative strategy to improve the quality of marginal grafts.

## Background

Organ transplantation provides a life-saving opportunity for patients with end-stage organ failure. However, the existing donor pool is far from meeting the ever-growing demand for transplantable organs. One approach to alleviate the shortage of suitable organs has been the expansion of the deceased donor pool by utilizing marginal organs from donation after circulatory death (DCD) (1) and extended criteria donors (ECD) (2). However, marginal grafts are vulnerable to ischemia and have not enough physiological reserves to tolerate ischemia-reperfusion injury (IRI) during transplantation (3), leading to an increased incidence of primary graft dysfunction (PGD) and delayed graft function (DGF) (4, 5). Therefore, improving the quality of marginal organs will be a significant strategy to increase the source of donors.

In the process of organ transplantation, the donor grafts will be deprived of blood supply during procurement and suffer from a long duration of ischemia when the grafts are preserved and transported. The deprivation of blood supply leads to consumption of energy storage, anaerobic metabolism, as well as disorders in cellular activities. Toxic metabolites accumulate during ischemia and will exacerbate the oxidative injury and inflammation after reperfusion (6). The restoration of blood flow following engraftment aggravates cell death *via* complex cellular cascades and further worsens the quality of grafts (7). Therefore, efforts are needed to improve the quality of marginal organs by alleviating the IRI during transplantation.

Static cold storage (SCS) is the most widely used method for organ preservation, but in recent years, machine perfusion has emerged as a promising alternative to SCS (8). Machine perfusion can continually perfuse the organs to mimic the physiologic condition. Grafts can restore metabolism and even resume their function during perfusion, especially in the normothermic environment with the supply of substrates and oxygen (9). Machine perfusion is proposed to reduce the IRI with an improvement of graft quality and prolong organ preservation time, as well as allow objective evaluation for the viability of grafts during preservation (10). Another highlight of machine perfusion is offering a pivotal opportunity to recondition the high-risk grafts during preservation (11).

Mesenchymal stem cells (MSCs) have attracted tremendous attention due to their immunomodulatory property and regenerative effects. Current literature has suggested the beneficial effects of MSCs or their secretory factors in cases of organ IRI and solid organ transplantation (12). However, the majority of administered MSCs in animals or humans would be entrapped in lungs and could not survive for a long time (13, 14). Also, the high dosage of MSCs would lead to microvascular obstruction (15).

MSCs therapy and machine perfusion may supplement each other if combined properly, as machine perfusion can provide a separate platform and time window for MSCs to recondition the isolated grafts *via* the immunomodulatory and regenerative effect (9). In this review, we presented an overview of the current literature regarding the application of MSCs during machine perfusion on solid organ transplantation.

## A Brief Introduction of IRI in Organ Transplantation

IRI is inevitable during organ transplantation. It occurs when the blood supply of grafts is stopped and restored. Hypoxia during ischemia leads to adenosine triphosphate (ATP) depletion and anaerobic metabolism, as well as subsequent disorders in membrane transport, calcium excretion, mitochondrial activity, and reactive oxygen species (ROS) turnover (16). Toxic metabolites accumulate during ischemia and induce further injury at the phase of reperfusion. For example, lactate, the production of anaerobic metabolism, leads to a drop in intracellular pH (17), while the accumulation of hypoxanthine can increase ROS production during reperfusion (18). The suddenly increased oxygen concentration during reperfusion results in a burst of ROS production, subsequently exacerbating the oxidative damage (17). The burst of ROS was validated to damage mitochondrial respiratory chain and metabolism enzymes further leading to more ROS production and impaired ATP production (19, 20). The excessive free radical also causes oxidative damage to the mitochondrial membrane and consequently increases mitochondrial permeability resulting in the release of pro-apoptotic factors to the cytoplasm (19). Inflammation is an important aspect of IRI. The danger-associated molecular patterns released by the injured cells could activate the innate immune responses *via* the Toll-like receptors (TLRs) and recruit immune cells (6). The activated immune responses initiate the production and release of inflammatory cytokines, and upregulation of endothelial adhesion molecules, which facilitates leukocyte adhesion and migration into grafts during reperfusion and further augments the inflammatory response (6, 21). The processive oxidative damage and inflammatory response ultimately lead to the activation of different cell death programs (apoptosis, necrosis, necroptosis, pyroptosis, and autophagy-associated cell death) (22). The pathophysiological process of IRI plays an important role in the deterioration of graft quality at the time of transplantation. Therefore, continuous efforts are necessary to alleviate the IRI of grafts during transplantation.

## Machine Perfusion: A Promising Technique for Organ Preservation

SCS is introduced as a standard approach for organ preservation—grafts are cooled down and transported in the “ice box” (23). Hypothermia can slow down metabolic activity and then alleviate the impact of ischemia on grafts during preservation. Consequently, the grafts can tolerate a short time of ischemia with maintained cell viability (24). However, the remaining metabolism still leads to progressive damage with the decreased energy stores, acidosis, and ROS production (25). As marginal organs are vulnerable to IRI, SCS seems unable to meet the high preservation requirements of marginal grafts due to the anoxic environment (26).

In the past decades, the expanding donor pool has fueled the interest in machine perfusion. The machine perfusion system consists of a pump that maintains continuous perfusion to the grafts through the vasculature until engraftment (27). Machine perfusion can be simply divided into hypothermic machine perfusion (HMP) at 0-8°C, subnormothermic machine perfusion (SNMP) at 20-34°C, and normothermic machine perfusion (NMP) at 35-38°C (24).

HMP can keep the organs in a low metabolic state. The dynamic perfusion in the HMP system allows the supply of substrates and removal of toxic metabolites produced during preservation. Besides, if perfusate were actively oxygenated, HMP can further fulfill the remaining metabolic demand, restore tissue energy reserves and reduce oxidative stress (28, 29). Therefore, it is expected to minimize the IRI and allow a longer preservation time for grafts. In 2009, a landmark study conducted by Moers et al. demonstrated the superior potential of HMP to protect the deceased-donor kidneys from IRI compared with SCS (30) and prompted more and more researchers to focus on the area of machine perfusion and organ preservation (31–33).

Compared with HMP, NMP can provide an approximately physiological condition where the organs are kept at body temperature. With the continual supply of nutrients and oxygen, the organs can maintain an active metabolism so that blood-based perfusate is always necessary for the effective delivery of oxygen (34). The closer physiological microenvironment is thought to provide extra benefits. Urbanellis et al. showed that NMP significantly improved early renal function and alleviated renal IRI during preservation compared to HMP and SCS (35). A randomized trial also demonstrated that NMP was associated with less graft injury and discarded organs, and longer preservation time than SCS (36). In addition, NMP enables the grafts to resume their function. For example, livers can produce bile and lungs allow gas exchange while ventilated. Consequently, assessment of the function and viability of grafts during preservation comes to reality. It may assist the clinicians to evaluate whether the grafts are suitable for transplantation, which is especially necessary for the marginal organs (37). Glucose, pH, and biliary bicarbonate have been suggested as the indicators of bile duct injury during NMP of livers (38), while left ventricular end-systolic elastance is a prognostic factor for heart transplantation (39). As a midway approach between HMP and NMP, SNMP is expected to take advantage of reduced oxygen demand under subnormothermic conditions and ensure sufficient metabolism of the grafts for viability assessment (24, 40).

More importantly, machine perfusion provides an organ-repairing platform where pharmacological, gene and stem cell therapy can be administered to recondition and repair the grafts (9). Numerous agents combined with machine perfusion have been tested in preclinical studies, such as urokinase (41), prostaglandin E1 (PGE1) (42), steroids (43), and siRNAs (44).

## The Features and Properties of MSCs

MSCs represent a heterogeneous population of multipotent stem cells, which are plastic adherent cells, express specific surface antigens and have the potential to differentiate into adipocytes, osteoblasts, and chondrocyte progenitors (45). MSCs can be isolated from many tissues, including adipose tissue, umbilical cords, and bone marrow. With few expressions of human leukocyte antigen, MSCs present with limited immunogenicity and are able to evade allogeneic immune response (6, 46). MSCs are well documented to exert antioxidant, immunomodulatory, and regenerative properties mainly by the direct interaction with adjacent cells and paracrine effects (12). The secretome of MSCs consists of cytokines, adhesion molecules, growth factors, and extracellular vesicles (EVs). EVs are membrane-packed vesicles including apoptotic bodies, exosomes, and microvesicles (MVs) (47). Containing a cargo of proteins and genetic materials, EVs participate in cell-to-cell communication *via* transferring the contents (48).

Both the immunoregulatory and regenerative roles make MSCs of great interest in ameliorating organ IRI (6). MSCs-derived exosomes can convert macrophages into anti-inflammatory phenotype, which releases immunosuppressive cytokines and regulates the T-regulatory phenotype (49, 50). Tryptophan is an essential amino acid necessary for T-cell proliferation. The indoleamine 2,3-dioxygenase secreted by MSCs could eliminate the tryptophan and subsequently affect the proliferation and apoptosis of T-cells (51, 52). Also, MSCs suppress TLR4-dependent activation of dendritic cells, leading to an inhibition of cytokine production and antigen presentation to T-cells (53). Furthermore, MSCs activate tissue repairing by releasing various growth factors such as vascular endothelial growth factor (VEGF), hepatocyte growth factor (HGF), fibroblast growth factor (FGF), keratinocyte growth factor (KGF), insulin-like growth factor-1, and stromal cell-derived factor-1α (6, 54).

## The Potential of MSCs in Organ Reconditioning

**Figure 1:**
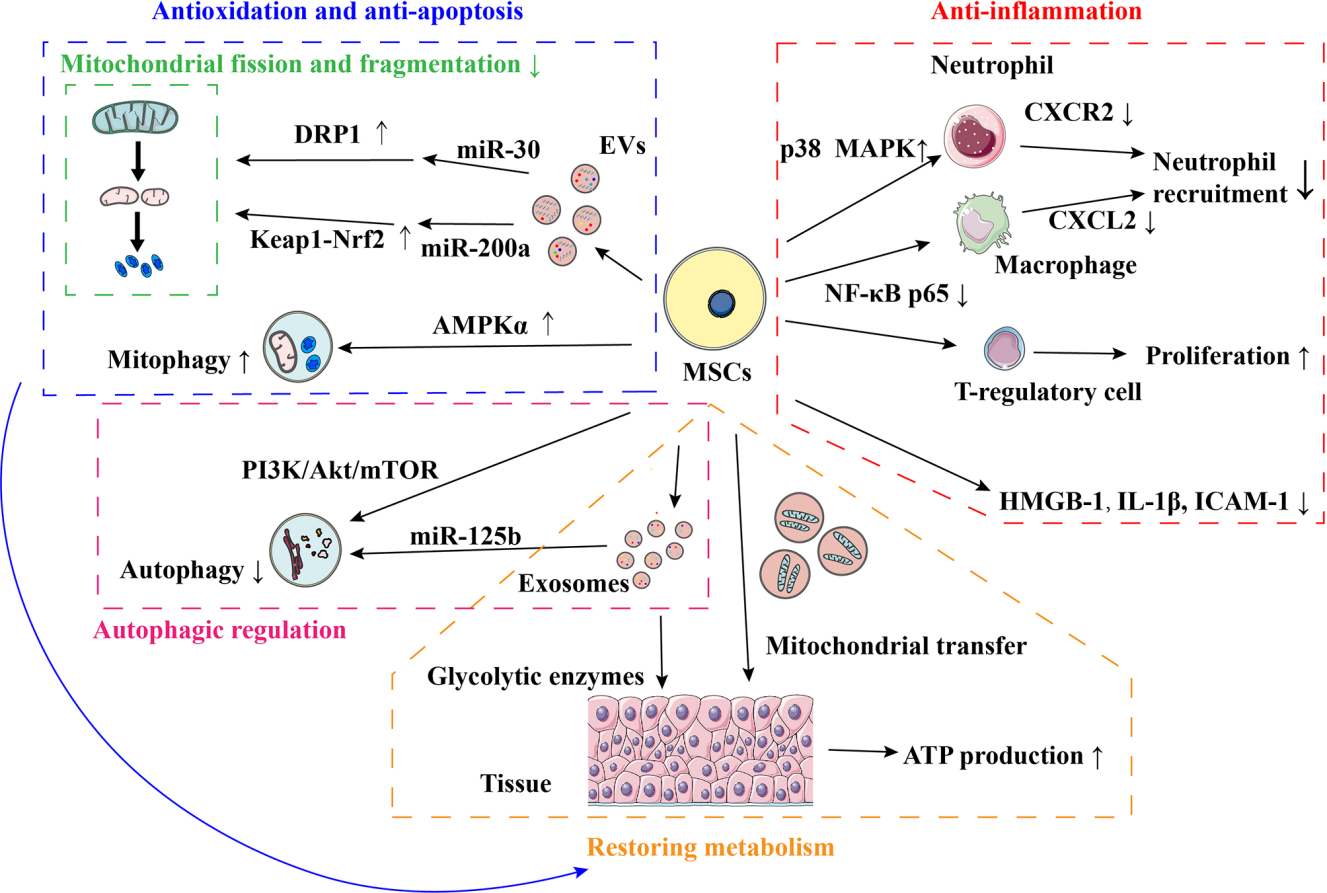
Mechanisms of MSCs in ameliorating organ IRI. AMPK, Adenosine monophosphate-activated protein kinase; ATP, Adenosine triphosphate; CXCL, CXC chemokine ligand; CXCR, CXC chemokine receptor; DRP1, Dynamin-related protein 1; EVs, Extracellular vesicles; HMGB-1, High mobility group box chromosomal protein-1; ICAM-1, Intercellular adhesion molecule-1; IRI, Ischemia-reperfusion injury; Keap1, Kelch-like ECH-associated protein 1; MAPK, Mitogen-activated protein kinase; NF-κB, Nuclear factor-Kb; Nrf2, NF-E2-related factor 2; IL, Interleukin; MSCs, Mesenchymal stem cells; PI3K, Phosphatidylinositol 3-kinase; mTOR, Mechanistic target of rapamycin.

Many studies have demonstrated the potential and underlying mechanism of MSCs to alleviate the organ IRI (**Figure 1**). Inflammation is an important aspect of IRI. MSCs can suppress the inflammatory response of renal IRI by inducing CD4^+^ Foxp3^+^ T-regulatory proliferation (55). MSCs can also improve hepatic IRI probably due to the suppressed transcription of inflammation-related genes within liver tissue, including high mobility group box chromosomal protein-1 (HMGB-1), interleukin-1β (IL-1β), and intercellular cell adhesion molecule-1 (ICAM-1) (56). As mentioned above, immune cell recruitment augments the inflammatory injury to the grafts at the reperfusion phase. Li et al. proved that MSCs ameliorated hepatic IRI predominantly *via* the inhibition of neutrophil migration and infiltration. They suggested MSCs not only reduced the neutrophil chemoattractant CXCL2 (CXC chemokine ligand-2) production in macrophages by suppressing nuclear factor κB (NF-κB) p65 phosphorylation but also promoted the p38 mitogen-activated protein kinase (MAPK) phosphorylation to decrease the CXCR2 (CXC chemokine receptor-2.) expression on the surface of neutrophils (57).

MSCs were found to alleviate the organ IRI by improving metabolic activity. Lai et al. suggested that glycolytic enzymes were present in MSCs-derived exosomes and could be transferred to the reperfused myocardium to prompt the glycolytic flux and ATP production. They believed that the rapid ATP production could help the initiation of cellular processes in cardiac tissue immediately following reperfusion (58). Mitochondrial dysfunction plays an important role in the pathophysiological process of IRI, leading to excessive ROS generation, impaired ATP production, and apoptosis. Tseng et al. observed that mitochondria were transferred from MSCs to neurons *in vitro* after oxidative insult through cell-to-cell contact. The transfer of mitochondria recovered the metabolic activity in neurons with the improvement of mitochondrial respiration, basal metabolic rate, spare respiratory capacity, proton leak, and ATP production (59). Mitochondria constantly undergo fission and fusion and would shift to the fission state in response to IRI, leading to mitochondrial fragmentation and cell apoptosis (60, 61). Gu et al. uncovered that the administration of MSCs-derived EVs (MSC-EVs) immediately after reperfusion suppressed mitochondrial fission and subsequently mitochondrial apoptotic pathways in rat model of renal IRI. The inhibition of mitochondrial fission was probably mediated by miR-30 contained in MSC-EVs (61). Cao et al. suggested that MSC-EVs prompted renal repair after IRI by targeting and restoring mitochondrial function. They found that MSC-EVs protected kidneys from oxidative insult by reducing mitochondrial fragmentation and normalizing membrane potential. The transfer of miR-200a from MSCs was likely to increase mitochondrial antioxidant defense and ATP generation by activating the Keap1 (Kelch-like ECH-associated protein 1)/Nrf2 (NF-E2-related factor 2) pathway (62).

Autophagy participates in the process in IRI. It can be activated by ischemia, hypoxia, and nutrient deprivation to degrade and recycle the cytosolic proteins and damaged organelles for ATP production and protein synthesis (63). Xiao et al. observed a significantly reduced autophagic flux and apoptosis in infarcted mouse hearts treated with MSCs. They suggested the transplantation of MSCs after myocardial infarction could suppress the autophagic flux and cell death partially by exosomal transfer of miR-125b. In rat model of acute lung IRI, Lin et al. also found that the treatment of MSCs strongly downregulated the autophagic signaling pathway (64). As a special type of autophagy, mitophagy sequesters and eliminates unhealthy mitochondria (20). Anzell et al. deemed that clearance of unhealthy mitochondria is vital to avoid oxidative injury and apoptosis. In mouse liver with IRI, MSCs were administered immediately after reperfusion. Zheng et al. observed that the hepatoprotective effect of MSCs was accompanied by reduced mitochondrial ROS production, suppressed mitochondrial fragmentation, decreased apoptosis, and restored ATP generation. They suggested that MSCs upregulated the PTEN (phosphatase and tensin homolog)-induced putative kinase 1-dependent mitophagy to control the mitochondrial quality by adenosine monophosphate-activated protein kinase α (AMPKα) activation.

In recent years, MSCs have been suggested to recondition the donor grafts during transplantation. Montanari et al. found the intravenous infusion of MSCs to rats after heterotopic heart transplantation led to an early improvement in cardiac function and subsequent reduction in ventricular remodeling, cardiac fibrosis, and apoptosis in transplanted hearts (65). On the other hand, brain-dead donor hearts preserved by solution supplemented with MSCs or their secretome resulted in an improvement of posttransplant cardiac contractility and caspase-independent apoptosis (66, 67), while the hypoxic precondition could further enhance the beneficial effects of MSCs-derived secretome on apoptosis, histopathology, inflammation and functional performance to donor hearts after transplantation (68). Heme oxygenase-1 (HO-1) has shown the potential to inhibit oxidative stress (69), suppress inflammation (70), and enhance the quality of MSCs (71). Yang et al. delivered HO-1-transduced MSCs and MSCs respectively to rats immediately after liver transplantation. They found the HO-1 transduction could augment the positive impact of MSCs on the recovery of microcirculation and energy metabolism in transplanted livers (72).

**Figure 2:**
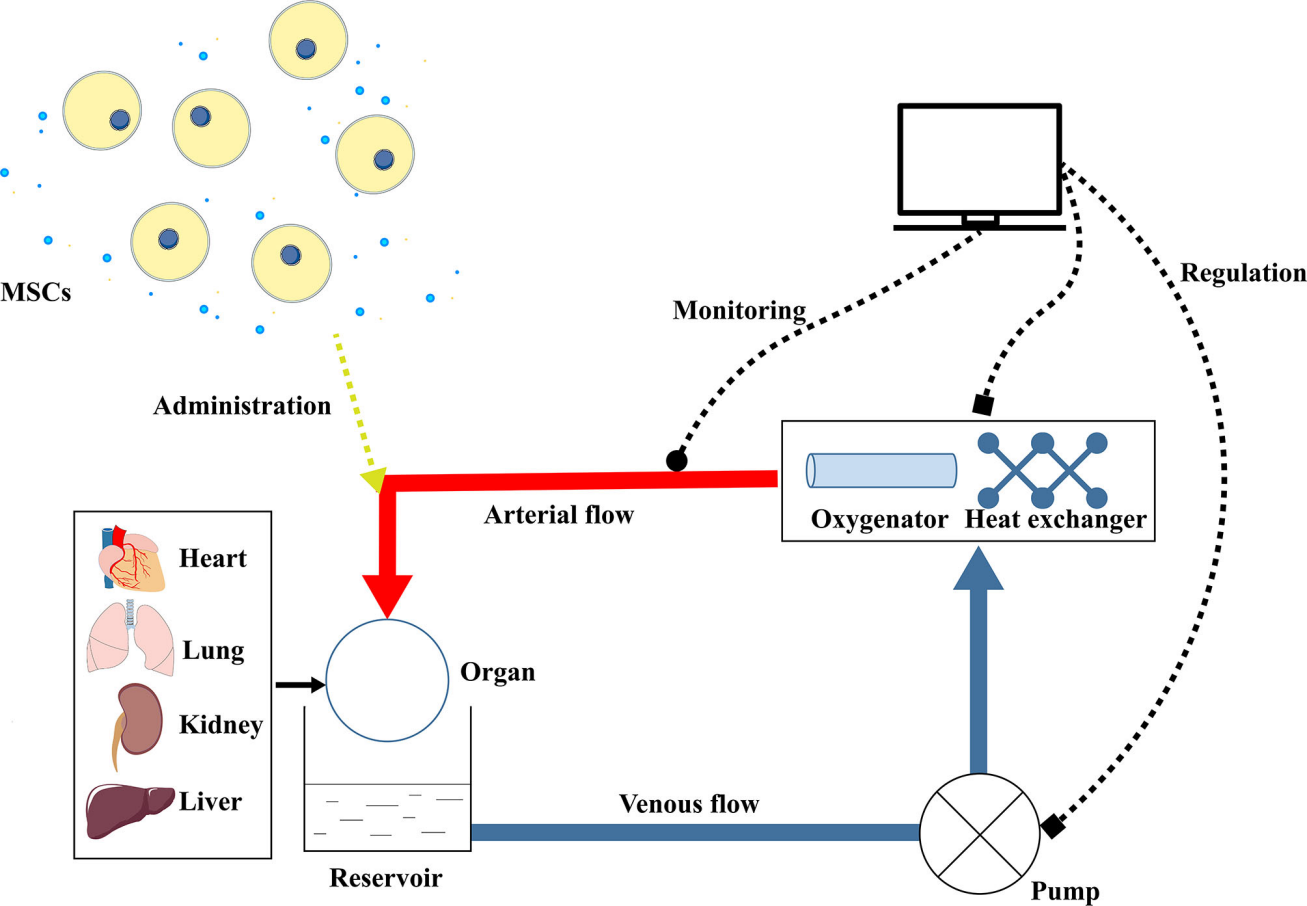
Schematic picture of MSCs combined with machine perfusion on organ preservation and reconditioning. The machine perfusion system consists of a pump, reservoir, oxygenator, and heat exchanger. The pump can maintain a continuous flow to the organ (heart, lung, kidney, or liver) through the vasculature. Recycled in the circuit, the perfusate is oxygenated by the oxygenator and kept at a certain temperature by the heat exchanger. The perfusate temperature, perfusion pressure, and perfusion flow rate are monitored and the pump and heat exchanger are accordingly regulated. During perfusion, MSCs or their secreted factors are administered in the perfusate to the isolated organ, which is expected to ameliorate the IRI and quality of the graft. IRI, Ischemia-reperfusion injury; MSCs, Mesenchymal stem cells.

MSCs are commonly infused through a peripheral vein to both animals and patients (73). But intravenous administration of MSCs or their secretome is facing the problem of entrapment in the lungs or absorption by other tissue with only a small proportion remaining in the target organs (13). Although a higher dosage may address this concern, the risk of microvascular embolism and side effects on other organs increase (74). Also, the infused MSCs have a lower long-term survival rate in the recipients (14). Machine perfusion provides an ideal platform for MSCs to directly recondition the target organs irrespective of the physiological barriers and adverse effects on other organs. Additionally, the amount of MSCs could be downregulated to avoid microvascular obstruction and MSCs could be protected from the whole immune system during perfusion, which guarantees their therapeutic effects on donor grafts. The combination of machine perfusion and MSCs is likely to have extra benefits to the grafts and is possible to convert the marginal organs to transplantable ones (**Figure 2**).

## Application of MSCs Combined With Machine Perfusion in Organ Transplantation

Recently, multiple efforts have been made to investigate the therapeutic effects of MSCs combined with machine perfusion on donated organs (**Tables 1** and **2**).

**Table 1:** Recent studies regarding the applications of MSCs during machine perfusion on organ transplantation.

References	Organs	Model	Perfusion type	Perfusate	Recondition time	Agents		Engraftment	Year
(75)	Lung	Human lungs rejected for transplantation	NMP	DME H-21 + 5% bovine serum albumin	4 h	Human BMMSCs		–	2014
(76)	Lung	Human lungs rejected for transplantation	NMP	DMEM without Phenol Red + 5% bovine serum albumin	6 h	Human BMMSC-MVs		–	2015
(77)	Lung	Porcine lungs	NMP	Steen solution + heparin + cefazolin +methylprednisolone	11 h	Human UCMSCs		–	2016



(78)	Lung	Murine DCD lungs	NMP	Steen solution + heparin + cefazolin + methylprednisolone	1 h	Human UCMSCs/UCMSC-EVs		–	2017



(79)	Lung	Rat lungs	NMP	–	2 h	MSC-EVs*		–	2019
(80)	Lung	Porcine lungs	NMP	Steen solution + heparin + cefazolin +methylprednisolone	10 h	Human UCMSCs		Orthotopic left single-lung transplantation	2019



(81)	Kidney	Rat DCD kidneys	HMP	Belzer solution	4 h	Rat AMSC-EVs		–	2017
(82)	Kidney	Human DCD kidneys	SNMP	Acellular medium	24 h	MSCs*		–	2018
(83)	Kidney	Porcine DCD kidneys	NMP	Williams’ Medium E + amoxicillin-clavulanate + albumin + pure red blood cells	6 h	Human AMSCs/BMMSCs		–	2019



(84)	Kidney	Porcine DCD kidneys	NMP	0.9% sodiumchloride + pure erythrocytes + albumin +sodiumbicarbonate + calciumgluconate + glucose +insulin + mannitol + creatinine + amoxicillin/clavulanate	6 h	Human AMSCs/BMMSCs		–	2020
(85)	Kidney	Porcine DCD kidneys	NMP	allogeneic erythrocytes + albumin + sodium bicarbonate + glucose + insulin + calcium gluconate + mannitol + creatinine	3 h	Human/porcine AMSCs		Autotransplantation	2020
(86)	Liver	Rat DCD livers	NMP	Krebs-Henseleit solution	2 h	Swine AMSCs		–	2018
(87)	Liver	Porcine DCD livers	HMP	University of Wisconsin solution	0.5 h	Human BMMSCs		–	2018
(88)	Liver	Rat DCD livers	NMP	DMEM/F12 + fetal bovine serum + penicillin–streptomycin solution + heparin + insulin + dexamethasone + fresh blood	8 h	Rat BMMSCs		–	2020
(89)	Liver	Rat DCD livers	NMP	DMEM/F12 + fetal bovine serum + penicillin–streptomycin solution + heparin + insulin + dexamethasone + fresh blood	8 h	Rat BMMSCs		–	2020
(90)	Liver	Rat DCD livers	NMP	DMEM/F12 + fetal bovine serum + penicillin–streptomycin solution + heparin + insulin + dexamethasone + fresh blood	4 h	Rat HO-1-modified BMMSCs/BMMSCs		Orthotopic liver transplantation	2020
(91)	Heart	Aged rat hearts	HMP	Custodiol solution	5 h	Rat BMMSC-CM	Heterotopic heart transplantation		2019

AMSCs, Adipose-derived mesenchymal stem cells; AMSC-EVs, Extracellular vesicles derived from AMSCs; BMMSCs, Bone marrow-derived mesenchymal stem cells; BMMSC-CM, Conditioned medium derived from BMMSCs; BMMSC-MVs, Microvesicles derived from BMMSCs; DCD, Donation after circulatory death; HMP, Hypothermic machine perfusion; NMP, Normothermic machine perfusion; MSCs, Mesenchymal stem cells; MSC-EVs, Mesenchymal stem cells-derived extracellular vesicles; SNMP, Subnormothermic machine perfusion; UCMSCs, Umbilical cord-derived mesenchymal stem cells; UCMSC-EVs, Extracellular vesicles derived from UCMSCs.

*The origin of MSCs was not mentioned in the article.

**Table 2:** The therapeutic effects of MSCs combined with machine perfusion on organ transplantation.

Reference	Organs	Outcomes								
		Function	Histopathology	Injury	Inflammation	Oxidative stress	Apoptosis	Metabolism	Growth factors	Others
(75)	Lung	↑AFC	–	–	–	–	–	–	–	–
(76)	Lung	↑AFC; lung compliance		↑Angiotensin-I	–	–	–	–	–	↑NO
		↓Tracheal pressure; PAR; PAP	↓Lung weight	↓Syndecan-1						
(77)	Lung	↑PaO_2_/FIO_2_; static lung compliance	–	–	↓IL-8	–	–	–	↑VEGF	–
(78)	Lung	↑Pulmonary compliance		–		–	–	–	–	–
		↓PAP	↓Edema		↓Neutrophil infiltration					
(79)	Lung	↓Total pulmonary	–	–	↑Genes		–	↑ATP	–	↑NO; hyaluronan
		vascular resistance			involved in resolution of inflammation and oxidative stress			↓Glucose; lactate		
(80)	Lung	↓Peak airway pressure	↓Edema; histologic acute lung injury scores	–	↑IL-4	–	↓Apoptosis	–	↑HGF	–
					↓IL-18; IFN-γ; TNF-α; T-cell infiltration					
(81)	Kidney	–	↓Renal damage score; bleb formation; tubular necrosis; tubular lumen obstruction	↓LDH	–	↓MDA;	–	↑Genes involved in cell energy metabolism pyruvate	–	↑Genes involved in membrane transport
								↓Glucose; lactate		
(82)	Kidney	–	–	–	↓Proinflammatory cytokines	–	–	↑ATP	↑EGF; FGF-2; TGF-α	↑Mitosis; PCNA
(83)	Kidney	–	–	–	–	–	–	–	–	–
(84)	Kidney	–	–	↓NGAL; LDH	↑IL-6; IL-8	–	–	–	↑HGF	–
(85)	Kidney	No observed significant difference between groups treated with or without MSCs								
(86)	Liver	↑Bile production	↓Sinusoidal space narrower; hepatocellular vacuolation	–	–	–	–	–	–	–
(87)	Liver	–	–	–	–	–	–	–	–	–
(88)	Liver	↑Bile production;				↑GSH			–	↑AMPK activation
			↓Histopathological score; vacuolar degeneration; hepatic sinusoid congestion; inflammatory cell infiltration; edema	↓ALT; AST; mitochondrial damage	↓MPO	↓MDA	↓Apoptosis	↓Lactate		↓JNK/NF-κB pathway
(89)	Liver	↑Bile production	↓Histopathological score; vacuolar degeneration; hepatic sinusoid congestion; inflammatory cell infiltration; edema	↓ALT; AST; ALP; mitochondrial damage	↓ICAM-1; VCAM-1; macrophage activation	–	↓Apoptosis	↓Lactate	–	↓vWF; ET-1
(90)	Liver	↓Bile duct injury; histopathological score;	–	↓ALT; AST; ALP; GGT;	↓Proinflammatory cytokines (IL-1β, IL-6, TNF-α); TLR4/NF-κB pathway-related molecules	–	–	–	–	↑Recipient survival time
										↓HMGB1
(91)	Heart	↑Cardiac function			↓Genes involved in inflammation, oxidative stress, apoptosis					↓Genes involved in PI3K/Akt pathway

AFC, Alveolar fluid clearance; ALP, Alkaline phosphatase; ALT, Alanine aminotransferase; AMPK, Adenosine monophosphate-activated protein kinase; AST, Aspartate aminotransferase; ATP, Adenosine triphosphate; EGF, Epidermal growth factor; ET-1, Endothelin-1; FGF-2, Fibroblast growth factor-2; GGT, Glutamyl transpeptidase; GSH, Glutathione; HGF, Hepatocyte growth factor; HMGB1, High mobility group box chromosomal protein-1; HO-1, Heme oxygenase-1; ICAM-1, Intercellular adhesion molecule-1; IFN-γ, Interferon-γ; IL, Interleukin; JNK, c-Jun N-terminal kinase; KGF, Keratinocyte growth factor; LDH, Lactate dehydrogenase; MDA, Malonaldehyde; MPO, Myeloperoxidase; NF-κB, Nuclear factor-κB; NO, Nitric oxide; PAP, Pulmonary artery pressure; PAR, Pulmonary artery resistance; PCNA, Proliferating cell nuclear antigen; PGE, Prostaglandin E; PI3K, Phosphatidylinositol 3-kinase; ROS, Reactive oxygen species; TGF-α, Transforming growth factor-α; TLRs, Toll-like receptors; TNF-α, Tumor necrosis factor-α; VCAM-1, Vascular cell adhesion molecule-1; VEGF, Vascular endothelial growth factor; vWF, Von willebrand factor.

## MSCs and Machine Perfusion for Lung Transplantation

Since both pulmonary vascular and bronchial trees are direct access to the entire parenchyma (9), MSCs can be administered *via* the bronchus or the vasculature. In NMP of porcine lungs, Mordant et al. reported that 50×10^6^ MSCs delivered through the pulmonary artery showed higher retention in the parenchyma and similar tolerance to the intrabronchial administration of the same dose (77). They also compared the impact of the bronchial fluid and perfusate (Steen fluid) harvested during NMP on the viability of MSCs *in vitro*. Equivalent to unadulterated Steen fluid, MSCs after 18-hour exposure to bronchial fluid presented lower viability than those exposed to the perfusate, suggesting bronchial fluid was not the ideal environment for MSCs and protective factors for the MSCs survival were released into the perfusate during perfusion (77). However, they didn’t compare the outcome of lungs exposed to MSCs under different administration routes. Lee et al. previously found that there was no difference between the intravascular and intrabronchial routes in the efficacy of MSCs on the isolated lungs injured by *E. coli* bacteria, which might be related to the paracrine effect of MSCs (92).

Additionally, concern about the microvascular embolism will be raised if MSCs are intravascularly administered during machine perfusion (15). The majority of MSCs administered in the perfusate retained in lungs within a few minutes and could be observed in both capillaries and the alveolar interstitium at the end of NMP (77, 80). Mordant et al. found that higher doses of MSCs were associated with higher retention of cells in the pig lungs. Also, 150×10^6^ MSCs delivered during NMP were well tolerated without change in pulmonary vascular resistance and showed significant improvement in PaO_2_/FIO_2_ and static lung compliance. However, the double dose of MSCs was associated with increased pulmonary vascular resistance without benefits to pulmonary physiology. Accordingly, the optimal dose was suggested to be 5×10^6^ MSCs per kilogram of animal weight (77).

Noncardiogenic pulmonary edema occurs in the early phase of PGD after lung transplantation (93). Alveolar fluid clearance (AFC) is defined as the ability of the lung to reabsorb the fluid in alveoli, which is dependent on an intact epithelial barrier (94). Accordingly, IRI may impair the integrity of epithelium and subsequent accumulation of fluid in alveoli may lead to pulmonary edema and reduced oxygenation (95, 96). In the human lungs rejected for transplantation, McAuley et al. demonstrated that the AFC could be normalized by MSCs in combination with NMP, partially mediated by KGF secretion (75). It was reported that exogenous KGF could increase the fluid transport capacity of the alveolar epithelium (97). In a model of acute lung injury, MSCs were proved to restore lung fluid balance by transferring KGF mRNA to the injured alveolar epithelium through the MVs (98). In line with previous studies, Gennai et al. found that the administration of MSCs-derived MVs (MSC-MVs) during NMP restored the AFC in a dose-dependent manner and decreased lung weight gain following perfusion. Additionally, the reduced perfusate level of syndecan-1 and the elevated level of angiotensin-I in the injured alveolus suggested a partial restoration of lung endothelium (76). Mordant et al. reported that the administration of MSCs during NMP could significantly decrease the perfusate level of IL-8 and increase the parenchymal concentration of VEGF (77), which was inversely correlated with alveolar epithelial damage (99). HGF has been shown to protect the integrity of endothelial junction and improve endothelial permeability (100, 101). Nakajima et al. suggested that HGF probably mediated the amelioration of pulmonary edema and lung injury after transplantation in the group treated with MSCs and NMP, since its concentration was high in both perfusate and lung tissue. Also, Nakajima et al. reported that MSCs administered during NMP could reduce the level of apoptosis, T-cell infiltration, and proinflammatory cytokines in lung tissue after transplantation. They inferred that the anti-inflammatory and anti-apoptotic effects of MSCs were pratially mediated by HGF (80), which could reduce the production of IL-12, IL-18, and tumor necrosis factor-α (TNF-α) (102), and inhibit apoptosis by regulating PI3K (phosphatidylinositol 3-kinase)/Akt and MAPK pathways (103).

Stone et al. found that NMP could improve pulmonary function and edema in murine models. Interestingly, MSCs and MSC-EVs could comparably and effectively enhance the protective and rehabilitative effects of NMP with significant improvement in pulmonary compliance and pulmonary artery pressure. The MSCs/MSC-EVs delivered to the lungs during NMP also attenuated neutrophil infiltration, pulmonary edema, and lung injury compared to NMP alone. They suggested that MSCs/MSC-EVs might present immunomodulatory and endothelial barrier-protective properties during NMP, *via* upregulating the anti-inflammatory molecules (IL-10, KGF, PGE2) expression, suppressing the activation of alveolar macrophages and invariant natural killer T cells, and mitigating neutrophil transendothelial migration (78). Besides, Lonati et al. found that MSC-EVs could induce the expression of various genes involved in anti-inflammatory response and resolution of oxidative stress in rat lungs during NMP. They showed that MSC-EVs could transfer hyaluronan into lung tissue and induce pulmonary production of hyaluronan during NMP (79). The transferred hyaluronan was primarily medium-high-molecular-weight, which was involved in the immunomodulatory and regenerative effects of MSCs (104). Similar to the study conducted by Gennai et al. (76), Lonati et al. found that the airway and hemodynamic parameters were significantly ameliorated in lungs treated with MSC-EVs during NMP, which might be attributed to the increased perfusate level of nitric oxide. Besides, the improvement of perfusate lactate and tissue ATP content in the group treated with MSC-EVs suggested the recovery of aerobic metabolism (79).

## MSCs and Machine Perfusion for Kidney Transplantation

The high risk of primary nonfunction and DGF hinders the adoption of marginal grafts in kidney transplantation. Fortunately, previous studies have demonstrated the beneficial effects of HMP on the quality of high-risk renal grafts (105). Thereafter, Gregorini et al. proved the protective effect of MSCs/MSC-EVs combined with HMP on rat DCD kidneys. 3×10^6^ MSCs were administered during HMP and detected in vessels, tubules, and interstitium of the kidneys at the end of 4-hour perfusion. The histologic evaluation showed that MSCs could significantly ameliorate the severe lesions (bleb formation, tubular necrosis, and tubular lumen obstruction) in DCD kidneys compared to those perfused only. Besides, genes involved in molecular transport, respiratory electron transport, and citric acid cycle were significantly up-regulated in the MSCs-treated group. They suggested that MSCs ameliorated the cellular metabolism and ischemic injury of DCD kidneys during HMP, as indicated by the increased pyruvate level and reduced perfusate level of lactate dehydrogenase, malonaldehyde (MDA), lactate, and glucose. More importantly, the MSC-EVs were found to ameliorate renal ischemic injury during HMP more effectively and more rapidly, which might be attributed to the prompt availability of MSCs mediators contained in EVs (81).

A does-effect study was conducted by Brasile et al. (82) to determine the optimal number of MSCs delivered to the discarded human kidneys during 24-hour SNMP. 25, 50, 75, 100, and 200×10^6^ MSCs were respectively administered during SNMP (32℃) and 100×10^6^ was suggested as the optimal dose which would not adversely affect the perfusion pressure, vascular flow, and oxygen consumption. The histologic examination demonstrated that the infused MSCs retained in the vasculature without migration to the renal parenchyma, which was quite different from the findings by Gregorini et al. (81). In their subsequent experiments, they showed that the addition of MSCs resulted in a more evident ATP storage, reduced perfusate level of proinflammatory cytokines, and increased synthesis of epidermal growth factor (EGF), FGF-2, and transforming growth factor-α in comparison to perfusion only. Furthermore, upregulation of cell proliferation in kidneys treated with MSCs during SNMP was observed. The increased synthesis of growth factors probably mediated the regenerative effect of MSCs on injured kidneys (82), as EGF and FGF-2 were proved to promote tubular regeneration (106), downregulate proinflammatory signaling (107), and attenuate renal IRI (108).

Moreover, a recent study has shown the feasibility of delivering MSCs during NMP to a porcine kidney. Kidneys were perfused with warm oxygenated blood-based perfusate for 7 hours and treated with different dosages of MSCs. Pool et al. showed that when the dosage was as high as 10×10^6^, a proportion of the MSCs could be detected in lumen of glomerular capillaries with intact structure after NMP, which indicated that the infused MSCs might remain viable and functional. Intriguingly, MSCs did not retain in the most neighboring glomeruli. The magnetic resonance imaging also showed an inhomogeneous distribution of MSCs in the perfused kidneys, which might be resorted to the anatomical difference of microvasculature leading MSCs to the path with less resistance during perfusion (83). In their subsequent study, Pool et al. investigated the alteration of renal function and factors secreted into the perfusate after the addition of MSCs during NMP. They found that the delivery of 10×10^6^ MSCs was not associated with very early renal function during NMP, but with increased levels of IL-6 and IL-8 in the perfusate. Besides, with fewer damage markers and more secretion of HGF in the perfusate, the perfused kidneys suffered less injury due to the addition of MSCs during NMP (84). Recently, the effect of MSCs combined with NMP on porcine kidneys after autotransplantation was investigated. Lohmann et al. found that the number of viable MSCs retaining in the transplanted kidneys dramatically dropped on postoperative day 14, and they failed to find the evidence of MSCs-induced recovery in transplanted kidneys, in line with the lack of improvement of renal function, early fibrosis markers and histology in the posttransplant phase (85).

## MSCs and Machine Perfusion for Liver Transplantation

Recently, Verstegen et al. reported the bioluminescent imaging of infused MSCs in porcine livers during HMP. A wide range and patchy distribution of infused MSCs in livers were observed throughout the 30-minute HMP, regardless of the arterial or venous infusion (87). However, lacking is strong evidence to illustrate the therapeutic effect of MSCs combined with HMP on DCD livers. As for the application of MSCs during NMP on DCD livers, Sasajima et al. found that the addition of MSCs during NMP could improve the bile production and ameliorate the sinusoidal space narrower and hepatocellular vacuolation in rat DCD livers (86).

Based on the promising results, a series of studies regarding the application of MSCs combined with NMP on rat DCD livers have been conducted by a research group. In the experimental group, Yang et al. delivered MSCs through the portal vein to livers at the initiation of NMP. During 6-hour NMP, MSCs were found to continually colonize in the hepatic sinusoid. Compared to livers solely submitted to NMP, livers treated with MSCs released less alanine aminotransferase and aspartate aminotransferase and produced bile more effectively. Histopathology evaluation also showed a significant improvement in MSCs-treated livers, regarding apoptosis, liver swelling, hepatic sinusoid congestion, cell vacuolar degeneration, and inflammatory cell infiltration. The level of MDA and myeloperoxidase was significantly decreased in livers treated with MSCs, with an increase of glutathione level. The MSCs-treated livers also presented less mitochondrial damage. Yang et al. confirmed in subsequent experiments that the inhibition of c-Jun N-terminal kinase/NF-κB pathway and the AMPK activation might mediate the positive effects of MSCs on DCD livers during NMP, as these two pathways were involved in oxidative stress. Also, MSCs were found to improve the microcirculation of DCD livers during NMP, *via* suppressing macrophage activation, reducing ICAM expression, and ameliorating epithelial cell damage (88, 89). Furthermore, in a subsequent study, the preserved DCD livers were used for orthotopic liver transplantation. In line with previous studies, Cao et al. found MSCs could improve the functional and histopathological performance of DCD livers after transplantation compared to those only exposed to NMP. Also, the recipient survival time was dramatically prolonged in the MSCs-treated group. Interestingly, MSCs significantly attenuated the inflammatory response in DCD livers after transplantation, as indicated by the reduced level of IL-1β, IL-6, and TNF-α, and decreased expression of HMGB1 and TLR4/NF-κB pathway-related molecules. Therefore, they suggested that MSCs could promote the protective effect of NMP on DCD livers (90). Cao et al. subsequently reported that HO-1 transduction into MSCs could further improve the beneficial effects of MSCs combined with NMP on DCD livers, regarding liver function, histopathology, inflammation, and recipient survival time after transplantation (90).

## MSCs and Machine Perfusion for Heart Transplantation

Hearts from the elderly have been regarded as a promising source of donor grafts (26). However, aging was associated with cardiac structural and functional deteriorations (109) and aged hearts were vulnerable to IRI (110). Recently, Korkmaz-Icöz et al. demonstrated HMP combined with MSCs-derived secretome could protect the donor hearts harvested from 15-month-old rats after prolonged storage. They found that the grafts harvested from the aged rats presented a significantly impaired left ventricular contractile function and relaxation compared with those from younger rats. Furthermore, HMP with perfusate supplemented with MSCs-derived secretome improved the posttransplant cardiac function of the aged grafts, *via* regulating the gene expression involved in apoptosis, inflammation, oxidative stress, and PI3K/Akt pathway (91). However, only one research has investigated the cardioprotective effect of machine perfusion with MSCs in donor heart preservation so far.

## Concerns and Future Perspectives

The fate of MSCs after delivery to grafts during machine perfusion is worth considering. The administered MSCs are under the influence of temperature, perfusion pressure, flow rate, perfusate, and graft. Sierra Parraga et al. previously investigated the impact of perfusate on the attached and suspended MSCs *in vitro*. They found that both the suspension condition and blood-based perfusate affected the survival rate of MSCs and their adhesion ability to endothelial cells. Cultured by the perfusate, the attached MSCs showed higher viability compared to MSCs in suspension. Besides, although the perfusate induced an increase in the secretion of inflammatory cytokines from adherent MSCs, the secretory profile of MSCs was unaffected (111). In an NMP system without an organ in the circuit, the number of MSCs in suspension decreased over time and only approximately 10% of the cells remained detectable after 6 hours of perfusion (83). Notably, the suspension status is expected to be transient as MSCs can retain in the organs during machine perfusion. However, the retention of MSCs varies among organs. Nakajima et al. and Mordant et al. showed the majority of MSCs administered in the perfusate retained in lungs within a few minutes (77, 80), while Brasile et al. found MSCs remained predominately in the perfusate instead of the perfused kidney (82). Pool et al. showed MSCs could travel through the kidney and stop circulating over time, but only a small proportion of MSCs could be detected in the kidney (83). Further comprehensive research may be encouraged to figure out the impacts of machine perfusion on the administered MSCs.

In addition, the higher dosage of MSCs is associated with higher retention of cells in lungs (77), which can also apply to other organs. The optimal dosage of MSCs administered during machine perfusion was suggested under the basis of acellular perfusate (77, 82). The blood-based perfusate is always necessary for NMP in the clinic with a higher viscosity than acellular perfusion solution. Therefore, the optimal dosage of MSCs in the NMP would be probably limited by the viscosity of blood-based perfusate to avoid alteration of hemodynamic parameters. Since the application of MSCs has a risk of malignant development and microvascular embolism (15, 112) and MSCs rely more on the paracrine effect, further research may be encouraged to focus on the application of cell-free therapy combined with machine perfusion on organ preservation.

Another concern for the application of MSCs during machine perfusion is the heterogeneity of MSCs, a result of different donors, tissue sources, culture methods, and individual cells within a colonial population. The heterogeneity leads to disparities in surface markers, proliferation, differentiation potential, and secretory profile of MSCs from different sources (113). Sierra Parraga et al. demonstrated that the response of human MSCs to the NMP conditions was different from that of porcine MSCs. Human MSCs had higher resistance to suspension condition and better adhesion to endothelial cells in perfusion fluid than porcine MSCs. Additionally, human MSCs incubated in perfusate showed higher metabolic activity of mitochondria but more ROS production compared to porcine MSCs. What’s more, the impact of cryoprotection on MSCs was different between the human and porcine sources regarding survival, proliferation, adherent capacity, ROS production, and metabolic activity (111). Wilson et al. suggested that the cell populations were insufficiently defined in many studies and the MSCs heterogeneity was likely to compromise the reproducibility as well as the clinical translation of those researches (113). In the present review, contradictory results were found in studies focusing on the application of MSCs and machine perfusion on organ transplantation, especially on kidney transplantation. Therefore, the heterogeneity of MSCs may in part account for the contradictory findings. Considering MSCs heterogeneity is unavoidable, future studies are encouraged to present more detail about the origin and identification of MSCs to ensure the comparability of studies in this field. Besides, a better understanding of the mechanisms of MSCs in improving the graft quality can help determine the acceptable degree of MSCs heterogeneity by quantifying the active ingredients in future studies or even clinical practice (113).

Most of the aforementioned studies shared a common limitation that the long-term effect of MSCs combined with machine perfusion on grafts after transplantation was not evaluated. The impact of such an innovative strategy on the postoperative outcome should be investigated in further studies.

## Conclusion

In conclusion, because of the ever-increasing demand for transplantable organs, enormous efforts have been made to expand the deceased donor pool. Mounting evidence has demonstrated the superiority of machine perfusion plus MSCs to improve the graft quality, especially the marginal organs. It is expectable for the further development of such an innovative strategy in organ transplantation and final application in the clinic.

## Author Contributions

JL, GS, SZ and PYZ contributed to the conception and design of the study. JL, QP, RY and KL searched the literature, wrote the manuscript, and created the figure and table. PZ and YZ participated in drafting the manuscript. PYZ, GS and SZ revised the manuscript. All authors read and approved the final manuscript.

## Funding

This research was supported by grants from Guangzhou Science and Technology Planning Project (No. 201804010067), Guangdong Science and Technology Planning Project (No. 2017ZC0064 and 2017A030303022), President Foundation of Nanfang Hospital, Southern Medical University (No. 2019c030), the Fellowship of China Postdoctoral Science Foundation (No. 2020M681559), and the National Natural Science Foundation of China (No. 82170274 and 82100410).

## Conflict of Interest

The authors declare that the research was conducted in the absence of any commercial or financial relationships that could be construed as a potential conflict of interest.

The reviewer QL has declared a shared affiliation, with no collaboration, with several of the authors JL, QP, PZ, YZ, and SZ, to the handling Editor at the time of review.

## Publisher’s Note

All claims expressed in this article are solely those of the authors and do not necessarily represent those of their affiliated organizations, or those of the publisher, the editors and the reviewers. Any product that may be evaluated in this article, or claim that may be made by its manufacturer, is not guaranteed or endorsed by the publisher.

## Glossary

AFC: Alveolar fluid clearance
ALP: Alkaline phosphatase
ALT: Alanine aminotransferase
AMSCs: Adipose-derived mesenchymal stem cells
AMSC-EVs: Extracellular vesicles derived from AMSCs
AMPK: Adenosine monophosphate-activated protein kinase
AST: Aspartate aminotransferase
ATP: Adenosine triphosphate
BMMSCs: Bone marrow-derived mesenchymal stem cells
BMMSC-CM: Conditioned medium derived from BMMSCs
BMMSC-MVs: Microvesicles derived from BMMSCs
CXCL: CXC chemokine ligand
CXCR: CXC chemokine receptor
DCD: Donation after circulatory death
DGF: Delayed graft function
DRP1: Dynamin-related protein 1
ECD: Extended criteria donors
EGF: Epidermal growth factor
ET-1: Endothelin-1
EVs: Extracellular vesicles
FGF-2: Fibroblast growth factor-2
GGT: Glutamyl transpeptidase
GSH: Glutathione
HGF: Hepatocyte growth factor
HMGB1: High mobility group box chromosomal protein-1
HMP: Hypothermic machine perfusion
HO-1: Heme oxygenase-1
Keap1: Kelch-like ECH-associated protein 1
ICAM-1: Intercellular adhesion molecule-1
IFN-γ: Interferon- γ
IL: Interleukin
IRI: Ischemia-reperfusion injury
JNK: c-Jun N-terminal kinase
KGF: Keratinocyte growth factor
LDH: Lactate dehydrogenase
MAPK: Mitogen-activated protein kinase
MDA: Malonaldehyde
MPO: Myeloperoxidase
MSCs: Mesenchymal stem cells
MSC-EVs: Mesenchymal stem cells-derived extracellular vesicles
MSC-MVs: Mesenchymal stem cells-derived microvesicles
mTOR: Mechanistic target of rapamycin
MVs: Microvesicles
NF-κB: Nuclear factor-κB
NMP: Normothermic machine perfusion
NO: Nitric oxide
Nrf2: NF-E2-related factor 2
PAP: Pulmonary artery pressure
PAR: Pulmonary artery resistance
PCNA: Proliferating cell nuclear antigen
PGE: Prostaglandin E
PGD: Primary graft dysfunction
PI3K: Phosphatidylinositol 3-kinase
PTEN: Phosphatase and tensin homolog
ROS: Reactive oxygen species
SCS: Static cold storage
SNMP: Subnormothermic machine perfusion
TGF-α: Transforming growth factor-α
TLRs: Toll-like receptors
TNF-α: Tumor necrosis factor-α
UCMSCs: Umbilical cord-derived mesenchymal stem cells
UCMSC-EVs: Extracellular vesicles derived from UCMSCs
VCAM-1: Vascular cell adhesion molecule-1
VEGF: Vascular endothelial growth factor
vWF: Von willebrand factor
